# Supplementary material for: Effect of patient and treatment factors on persistence with antihypertensive treatment: A population-based study
Source: PLoS One. 2021 Jan 15;16(1):e0245610. doi: 10.1371/journal.pone.0245610 (PMC7810514; doi:10.1371/journal.pone.0245610)
Supplement: S1 Table — (DOCX) [file pone.0245610.s001.docx]

**S1 Table. Sensitivity analyses: proportion of persistent antihypertensive users and median time to discontinuation according to the initially prescribed type of therapy when applying gaps of 30, 60 and 90 days.**

| **Type of therapy** | **n** | **n (%) of persistent users** | **Median time to discontinuation (days (CI 95%))** |
| --- | --- | --- | --- |
| ***Gap of 30 days*** |  |  |  |
| **Monotherapy** | 14,948 | 207 (1.4%) | 37 (32-40) |
| **Fixed combination** | 2,919 | 37 (1.3%) | 49 (45-51) |
| **Free combination** | 1,995 | 92 (4.6%) | 58 (57-59) |
| **Total** | 19,862 | 336 (1.7%) | 46 (44-47) |
| ***Gap of 60 days*** |  |  |  |
| **Monotherapy** | 14,948 | 6,125 (41.0%) | 208 (200-217) |
| **Fixed combination** | 2,919 | 1,262 (43.2%) | 236 (214-257) |
| **Free combination** | 1,995 | 1,026 (51.4%) | - |
| **Total** | 19,862 | 8,413 (42.3%) | 228 (221-236) |
| ***Gap of 90 days*** |  |  |  |
| **Monotherapy** | 14,948 | 8,738 (58.5%) | - |
| **Fixed combination** | 2,919 | 1,695 (58.1%) | - |
| **Free combination** | 1,995 | 1,285 (64.4%) | - |
| **Total** | 19,862 | 11,718 (59.0%) | - |

Abbreviations: n, number; %, percentage; CI, Confidence Interval.
