# Supplementary material for: Effect of patient and treatment factors on persistence with antihypertensive treatment: A population-based study
Source: PLoS One. 2021 Jan 15;16(1):e0245610. doi: 10.1371/journal.pone.0245610 (PMC7810514; doi:10.1371/journal.pone.0245610)
Supplement: Attachment — Submitted filename: Response To Reviewers.doc [file pone.0245610.s003.doc]

**EFFECT OF PATIENT AND TREATMENT FACTORS ON PERSISTENCE WITH ANTIHYPERTENSIVE TREATMENT: A POPULATION-BASED STUDY** (PONE-D-20-09984)

First, we would like to thank the academic editor and the reviewers for the provided comments, which have been very useful to improve the last version of the paper. The modifications made appear highlighted in the marked-up copy of the manuscript and are integrated in the unmarked version. Next, we explain the modifications applied based on the received letter:

*The points raised by the reviewers are adequately addressed. However, the following sentence does not seem to be correct: "In the latter case, for assessing the effect of polypharmacy on persistence, a relationship with inconsistent findings to date [8, 18]." As the is no copy-editing provided by PLOS One, the text must be copy-edited by the authors. I would like to ask you to correct this sentence and to very carefully re-read your article and copy-edit it. Thank you.*

Answer from authors:

Thank you for the comment. As requested, the sentence has been corrected: *“Moreover, the availability of information on other pharmacological treatments would have been useful for assessing the effect of polypharmacy on persistence, a relationship with inconsistent findings to date [8, 18]. “*

Moreover, the manuscript has been carefully re-read and some modifications have been introduced in order to improve its writing, wording and understanding.
